# Expression of Myosin 5a splice variants in murine stomach

**DOI:** 10.1111/nmo.14162

**Published:** 2021-05-03

**Authors:** Josephine A. Carew, Vivian Cristofaro, Nicolas A. Siegelman, Raj K. Goyal, Maryrose P. Sullivan

**Affiliations:** 1 VA Boston Healthcare System, Boston, MA, USA; 2 Harvard Medical School, Boston, MA, USA

**Keywords:** neurotransmission, peripheral nerve, protein splice variants, stomach, unconventional myosin

## Abstract

**Background:**

The motor protein, Myosin 5a (Myo5a) is known to play a role in inhibitory neurotransmission in gastric fundus. However, there is no information regarding the relative expression of total Myo5a, or of its alternative exon splice variants, across the stomach. This study investigated the differential distribution of Myo5a variants expressed within distinct anatomical regions of murine stomach.

**Methods:**

The distribution of Myo5a protein and mRNA in the stomach was assessed by immunofluorescence microscopy and fluorescent in situ hybridization. Quantitative PCR, restriction enzyme analysis, and electrophoresis were used to identify Myo5a splice variants and quantify their expression levels in the fundus, body, antrum, and pylorus.

**Key Results:**

Myo5a protein colocalized with βIII-Tubulin in the myenteric plexus, and with synaptophysin in nerve fibers. Total Myo5a mRNA expression was lower in pylorus than in antrum, body, or fundus (*p* < 0.001), which expressed equivalent amounts of Myo5a. However, Myo5a splice variants were differentially expressed across the stomach. While the ABCE splice variant predominated in the antrum and body regions, the ACEF/ACDEF variants were enriched in fundus and pylorus.

**Conclusions and Inferences:**

Myo5a splice variants varied in their relative expression across anatomically distinguishable stomach regions and might mediate distinct physiological functions in gastric neurotransmission.

## 1 | INTRODUCTION

The stomach is comprised of several anatomical regions, which are specialized to perform distinct yet coordinated functions, including reciprocating smooth muscular activity patterns that are essential for gastric motility. These patterns are neurally mediated, both through autonomic innervation (vagus and splanchnic nerves) and motor nerves within the myenteric plexus. As in brain neurons,^1–3^ enteric neurotransmitter-containing synaptic vesicles and the enzyme n-Nos, which synthesizes the neurotransmitter nitric oxide, rely on the actin-based motor protein Myo5a for short-range transport to and tethering at the presynaptic membrane. Not only has the presence of Myo5a in peripheral and enteric nerves been confirmed by immunohistochemistry,^4,5^ but a role for Myo5a in nitrergic smooth muscle relaxation has been demonstrated in visceral organs including the stomach, by functional studies.^6,7^

Myo5a is an unconventional motor protein composed of a dimerized pair of heavy chains, each organized into distinct functional domains. Near the N-terminus is an actin-binding and ATP-hydrolyzing region typical of myosin motors, followed by a neck segment with six binding sites for light chains including the calcium-sensing protein, calmodulin. Succeeding this are three coiled-coil regions which comprise the dimerization interface, and which partially overlap segments of alternatively expressed exons that may be arranged in a variety of configurations. At the C-terminus is the globular tail domain (GTD), which facilitates direct interactions between the motor and its cargos.^8–12^

The alternative exons of Myo5a in the medial tail region are consecutively lettered A, B, C, D, E, and F according to their order of appearance in the Myo5a gene. In the Myo5a protein, exons A, C, and E are present in all known splice variants, while exons B, D, and F seem to be expressed in a tissue-restricted manner.^8,9,13,14^ That distinct alternative exon configurations confer on Myo5a the ability to fulfill specialized transport functions by permitting interactions with key molecular cargos and/or their accessory protein partners has been established through comparison of the Myo5a variants expressed in brain neurons and skin melanocytes. Brain Myo5a includes exon B, which permits interaction with the accessory protein Dynll2 and the enzyme n-Nos, facilitating short-range transport of the latter to the presynaptic membrane^11,15^ but it lacks exons D and F. On the other hand, melanocyte Myo5a features exon F, which binds melanophilin, an accessory protein that couples Myo5a to the small GTPase, Rab27a, a component of the melanosome membrane. This interaction is essential for efficient melanin transport in melanocytes.^13,14,16,17^

Myo5a is known to be expressed throughout the body in neural tissue as well as other cell types specialized for secretion. Nonetheless, little is currently known regarding the variants of Myo5a and their operative role(s) within visceral organs. We addressed this information gap by investigating Myo5a mRNA expression in the antrum, body (corpus), fundus, and pylorus of stomach, using reverse transcriptase real-time PCR (RT-PCR) and restriction analysis to reveal relative abundance and linear exon configurations within the variable region. Gastric Myo5a consisted of several distinct splice variants, which were differentially distributed across the anatomical regions of stomach. Fluorescent in situ hybridization of pylorus suggested that Myo5a mRNA synthesis was largely confined to the myenteric plexus, while immunohistochemistry identified Myo5a protein not only in the plexus, but also throughout axons/nerve termini coursing within the muscular layers.

## 2 | MATERIALS AND METHODS

### 2.1 | Animals

All animal procedures were approved by the Institutional Animal Care and Use Committee of the VA Boston Healthcare System. Male mice (*Mus musculus* C57/BL6J) obtained from Jackson Labs were euthanized between 12 and 16 weeks by CO_2_ asphyxiation. Stomachs were removed, placed in cold physiological solution, and opened by longitudinal incision along the lesser curvature. After the gastric contents were flushed out, the mucosa was separated from the entire stomach with aid of a stereomicroscope. The borders of the pylorus were defined distally by the distinct change from villous to glandular mucosa at the pyloroduodenal opening and proximally by the sulcus intermedius. Tissue for the antrum was taken between the sulcus intermedius and the incisura angularis. The fundus was easily distinguished by its translucent appearance and defined ridge of mucosa at its border with the gastric body. The remaining tissue in the midportion of the stomach was trimmed distally to unequivocally delineate the region of the gastric body.

### 2.2 | Laser scanning confocal microscopy

Longitudinal muscle-myenteric plexus (LMMP) whole mount preparations were isolated from the gastric fundus by removing the mucosa, submucosa and circular layers using a dissecting microscope. LMMP was fixed with 4% paraformaldehyde (1 h, 4°C) and permeabilized by adding 0.25% Triton X-100 to the blocking solution. Rabbit anti-Myo5a (Sigma M4812 LF-18, hereafter referred to as LF-18, at 1:300) and mouse anti-βIII-Tubulin (Abcam Tu-20, ab7751, 1:300) primary antibodies were added simultaneously to preparations and incubated for 48 h at 4°C. After extensive washing in PBS, LMMP was incubated for 48 h in secondary antibodies; respectively, donkey anti-rabbit Alexa Fluor-488 (Invitrogen, A32790) and goat anti-mouse Alexa Fluor-568 (Invitrogen, A11031), each at 1:2000.

Specimens from the pyloric region were embedded in OCT compound and frozen in dry ice. Transverse sections (14 μm) were cut on a cryostat, fixed in cold acetone (10 min, −20°C), and subsequently blocked in phosphate-buffered saline (PBS) supplemented with 5% donkey serum and 0.1% Triton X-100. Sections were incubated with rabbit anti-Myo5a antibody (LF-18, 1:300) overnight at 4°C, followed by the anti-rabbit Alexa Fluor-488 secondary antibody (1:2000, 2 h, room temperature). After extensive washing, sections were incubated with mouse anti-synaptophysin antibody (Abcam Sy38, ab8049, 1:50 overnight at 4°C) followed by the anti-mouse Alexa Fluor-568 secondary antibody (1:2000, 2 h, room temperature).

Longitudinal muscle-myenteric plexus specimens and tissue sections were visualized on a confocal laser scanning microscope (Zeiss LSM 720) using a 40× objective. High-resolution images were acquired sequentially at each excitation wavelength to avoid spectral bleed-through. Control assays were performed in which each primary antibody was replaced with serum from the same species.

### 2.3 | RNA extraction and RT-PCR

Stomach tissues stripped of mucosa were stabilized by immersion in RNA-later solution (Sigma-Aldrich). Total RNA was purified using the Qiagen RNeasy Plus Mini kit using the manufacturer’s protocol, following tissue disruption in buffer RLT plus ß-mercaptoethanol with a 5 mm stainless steel bead, by two cycles of 3 min each at 30 Hz in the Tissue Lyser II apparatus (Qiagen). Total RNA concentration and purity were determined by ultraviolet spectrophotometry.

Aliquots of total RNA were converted to cDNA using the SuperScript IV VILO mastermix and then used for reverse transcriptase real-time PCR (RT-PCR) with universal PCR mastermix and TaqMan primer/probe sets for myo5a and for 18S ribosomal RNA as an internal control (Hs99999901_s1). Myo5a reagents (Mm01146036_m1, Mm01170524_m1, Mm01146039_m1, and Mm01146042_m1) were directed respectively against exon B, exon F, the junction between exons C and E (found only in variants lacking exon D), and the GTD (common to all variants), of murine Myo5a reference sequence, NM_010864.2, which carries the alternative exon arrangement ABCEF. Additional assays for n-Nos (Mm01208059_m1), Dynll2 (Mm00471607_m1), Syp (Mm00436850_m1), and alpha synuclein (Snca, Mm01188700_m1) were also used. All reagents were obtained from Thermo-Fisher Scientific. Reactions were performed on an Applied Biosystems QuantStudio 6 Flex instrument under a standard protocol (40 amplification cycles of 15 s at 94°C and 60 s at 60°C) in triplicate, normalized to triplicate internal control determinations, and analyzed by the ΔΔCt method. Stomach regions from three (antrum, pylorus) or four (body, fundus) C57/Bl6 animals were analyzed. Control assays were performed without added cDNA.

### 2.4 | Standard PCR and restriction digestion

cDNA prepared as described above was used in PCR as template with primer pair 1, which spans the alternative exon region, having its forward primer within exon A sequence and reverse primer within the GTD. Twenty-five cycles were performed in an Eppendorf Mastercycler using Taq PCR mastermix, and products were purified with Qia-quick PCR-purification kit (both from Qiagen), prior to nested reactions with internal primer pairs 2 and 3. Primer pair 1: forward, GCAGAAACTGAAGACATTGCAC; reverse, ATTCTGGCGAGACGTGTT. Primer pair 2: forward, GTCCTCATCTTGAGGTCGCA; reverse, TCAGCCGGGTGATCTCATGCTGCAGG. Primer pair 3: forward GTCCTCATCTTGAGGTCGCA; reverse TGATACTTCTTAGGGTCATCTGC. Products were electro-phoresed on 2% Metaphor agarose (Lonza Biologics) containing Sybr-green in Tris/acetate/EDTA buffer. Fragment mass estimation before and after digestion with restriction enzymes Sty I or Ban II (New England Biolabs) was done by comparison to double-stranded DNA standards (Thermo-Fisher). Gels were digitized and analyzed on an Amersham Imager 600 (General Electric) at CCD resolution 2048 × 1536, using Imager 600 software.

### 2.5 | RNA in situ hybridization

Isolated pylorus was embedded in OCT compound and frozen in dry ice. Sixteen micrometer sections were cut on a cryostat, fixed in 4% paraformaldehyde solution, and then probed for mRNA encoding murine βIII-Tubulin (Mm-Tubb3-C3) or myosin 5a (Mm-Myo5a-C2) using the RNAscope Multiplex Fluorescent V2 kit (ACD Bio) according to the manufacturer’s protocol for fresh-frozen tissue. Sections were also tested with a negative control probe for bacterial Dap-B gene and the positive control probes, murine PolR2a and Pp1B (not shown). High-resolution images were acquired by confocal laser microscopy at 40× magnification by sequential scanning at each excitation wavelength (Zeiss LSM 710, Zen software).

### 2.6 | Data analysis

Differences in expression among stomach regions detected by RT-PCR were analyzed by one-way ANOVA followed by Tukey’s multiple comparison test. Differences in band intensity on the analyzed images from agarose gel electrophoresis were determined by Student’s *t*-test. Data are presented as mean ± SD, and *p* < 0.05 was considered significant.

## 3 | RESULTS

### 3.1 | Myo5a is expressed in gastric nerves

In LMMP preparations, Myo5a was expressed within the myenteric plexus, as confirmed by βIII-tubulin immunoreactivity. Robust Myo5a immunoreactivity was detected within a network of cells, with staining throughout the cell bodies and in the interconnecting fibers (Figure 1A).

Myo5a immunoreactivity was also detected in varicose fibers coursing throughout gastric tissue running parallel to circular (Figure 1B) and longitudinal smooth muscle (not shown). Here within the muscular layers, expression of Myo5a colocalized extensively with synaptophysin, a marker of synaptic vesicles. The distribution pattern of Myo5a in enteric ganglia and nerve fibers running through the smooth muscle layers is consistent with the neuronal origin of Myo5a expression in gastric tissue.

The specificity of the M4812 LF-18 antibody for Myo5a was confirmed using a recombinant peptide derived from human Myo5a encompassing the epitope in the GTD, against which the antibody was raised (Figure S1). The LF-18 antibody strongly identified the peptide. In addition, the presence of the peptide reduced the ability of LF-18 antibody to recognize at least two protein bands of antrum and pylorus extracts corresponding to the reported molecular weight of Myo5a.

Furthermore, when fluorescent in situ hybridization (RNAscope) was done in pylorus to identify the site of Myo5a mRNA synthesis, intense punctate staining was confined to the myenteric plexus, which was identified based upon its location between the longitudinal and circular muscular layers. The mRNA for the neural marker, βIII-Tubulin, was also concentrated within the myenteric plexus (Figure 2). A similar expression pattern was obtained for RNAscope of fundus (not shown). These data indicated that mRNA analysis done on the stomach regions primarily reflects Myo5a transcription in neural tissue.

### 3.2 | Myo5a mRNA expression across stomach

The major stomach segments of mature C57/Bl6 mice were assessed for relative expression of the Myo5a gene using a TaqMan assay targeting the Myo5a GTD and normalized using the internal control, 18S ribosomal RNA (Figure 3). The antrum, body, and fundus showed approximately equivalent total Myo5a expression. However, pylorus expressed significantly less Myo5a overall than did any other region (all *p* < 0.001). This conclusion was confirmed by assays of fundus versus pylorus from additional C57/Bl6 mice, using a gene expression assay targeting a different exon/exon boundary in the Myo5a GTD, and comparing 18S rRNA versus beta-actin as the endogenous control (Figure S2).

A pattern similar to that of pan-Myo5a-GTD was seen for expression of the Syp and Snca genes, two markers of nerve varicosities. With the sole exception of Syp expression in the body, expression of both Syp and Snca were significantly higher in fundus, body, and antrum, compared with pylorus. Because Syp and Snca are abundant presynaptic varicosity proteins, their extents of expression may be considered indicators of the number of nerve termini present. That the patterns of Myo5a, Syp, and Snca expression paralleled one another suggested that the antrum, body, and fundus regions possessed a roughly equivalent number of nerve termini, which was greater than that of the pylorus.

### 3.3 | Expression of Myo5a alternative exons in stomach

Real-time PCR assays to investigate the relative extents of expression of exons B and F were also performed (Figure 3). These differed even in regions demonstrating equivalent overall Myo5a expression. All four regions expressed exon B, but notably antrum and body expressed significantly more exon B than did either fundus or pylorus (*p* < 0.001), though the antrum versus body and the fundus versus pylorus were not significantly different from one another.

Additionally, the distribution pattern of exon F throughout the stomach differed from that of exon B. For example, pylorus expressed slightly less exon F-containing variants than fundus or antrum (both *p* < 0.05), but not less than body. In addition, body was found to express less exon F-containing Myo5a mRNA than either antrum or fundus (*p* < 0.05 and *p* < 0.01, respectively).

Using the exon D-less assay, antrum demonstrated a significantly higher level of variants that lacked exon D, than did fundus. Since the overall expression of pan-Myo5a was not significantly different between them, it follows that antrum had lower expression of variants with exon D but higher expression of variants excluding exon D, than did fundus (*p* < 0.001). Differential expression of variants lacking exon D was not detected in comparisons between other stomach regions.

### 3.4 | n-Nos expression pattern across the stomach.

Myo5a exon B binds the accessory protein Dynll2,^11,15,18^ which in turn binds to n-Nos,^19^ forming a tripartite complex that, upon stimulation, permits translocation of n-Nos to the varicosity membrane as a prerequisite for its activation and the elaboration of the inhibitory neurotransmitter, nitric oxide. To examine expression of Dynll2 and n-Nos, additional TaqMan assays for these genes of interest were conducted. The n-Nos expression pattern across the stomach broadly reflected that of exon B. Antrum and body had the highest n-Nos expression at the mRNA level and did not differ significantly from one another. Conversely, pylorus demonstrated the lowest n-Nos expression, which was significant compared to the other areas (all *p* < 0.001). The n-Nos expression of fundus was intermediate between that of antrum/body and of pylorus and statistically different from both, being somewhat lower than antrum (*p* < 0.05) but higher than pylorus (*p* < 0.001). In contrast, Dynll2 expression was not found to differ significantly across stomach regions, perhaps reflecting its status as a hub protein which interacts with numerous partner proteins, among which Myo5a is but one (reviewed in Rapali et al.^20^).

### 3.5 | Alternative Myo5a exon configurations in stomach regions

To examine the linear arrangements of alternative exons comprising distinct splice variants, standard PCR and restriction enzyme analyses were performed on cDNAs from the stomach regions. The configuration of variants including exons B and D was examined in nested PCR products obtained with primer pair 2, which amplified the region from the distal exon A to the center of exon E and thus encompassed exons B and D, if present. Exon B introduces a Sty I restriction site, while exon D introduces a Ban II restriction site. In Figure 4 panel A, the undigested PCR products from replicate stomach regions, and their Sty I and Ban II digestion fragments, were compared by agarose gel electrophoresis.

Two PCR products were obtained for each region, and some of the smaller product could be converted into fragments of 299 and 50 bp (not visible) by Sty I, while remaining refractory to Ban II, thus representing configuration ABCE. A fraction of this product was resistant to Sty I, although enzyme was present in excess and the digestion time was long, suggesting that the nearly identically sized fragment (ACE) was represented to a lesser extent.

Inversely, the larger PCR product was resistant to Sty I but cleaved by Ban II, confirming that it lacked the 9 bp exon B but contained the 81 bp exon D, in agreement with its undigested mass. The expected doublet of 221/200 bp Ban II digestion fragments was resolved in digest lanes; thus, the larger product typified configuration ACDE.

A graphical representation of the electrophoretic data is shown in Figure 4B. For all stomach areas, the ABCE/ACE product was significantly more abundant than the ACDE fragment (all *p* < 0.001). Antrum and body, while not significantly different from one another, expressed more ABCE/ACE than did fundus (*p* < 0.001) or pylorus (*p* < 0.05). Conversely, fundus and pylorus expressed more ACDE than did antrum or body, which was apparent both before and after Ban II digestion (all *p* < 0.001), although comparisons between antrum and body, and between fundus and pylorus, were not significantly different.

In all regions, exon B and exon D were found almost exclusively in separate transcripts. Whether the ACE and ACDE configurations existed independently, or were part of larger arrangements including the 75 bp exon F, was next addressed by digestion of nested PCR performed with primer pair 3, which also spans the alternative exon region, but has its reverse primer located entirely within exon F and thus can amplify only those variants.

Figure 5A shows the exon F-containing products obtained from the stomach regions of a single C57/Bl6 animal, along with their Sty I and Ban II digestion patterns. Two products were obtained from each region, the larger and more abundant of which was of mass sufficient to include exon D. Again, exon B sequence could not be resolved by mass but was expected to introduce a Sty I motif. However, failure of Sty I to cleave either product from any region suggested that no exon F containing product included alternative exon B. That the Sty I restriction enzyme had been functional in this assay was confirmed by the parallel digestion of antrum nested PCR 2 product with this enzyme, shown in the lanes on the far right of Figure 5A. Ban II digestion was expected to cleave the larger exon D-spanning product into a doublet of 275/219 bp, and to release a minor digestion fragment from the smaller PCR product, while reducing the parent fragment size(s). The quantified results are graphed in Figure 4B and suggest that nearly all exon F in stomach occurs in a co-linear arrangement with exon D. This conclusion is supported by Ban II digestion of all PCR3 reactions from the stomachs of regions from additional animals shown in Figure 4C and graphically represented in Figure 4D. The larger and more abundant PCR3 product generated, which comprised about 90% of Myo5a transcripts with exon F, also included exon D. The identical pattern of these PCR and digestion products suggested that exons B and F did not occur together, although exons D and F did. Therefore, the preponderance of exon F-containing Myo5a transcripts across all stomach regions was found in configuration ACDEF, with a minor amount in configuration ACEF.

## 4 | DISCUSSION

This study identified the differential distribution of Myo5a in defined regions of mouse stomach and systematically characterized the regional expression of Myo5a splice variants. Like the transcripts of approximately three-quarters of all mammalian genes, Myo5a mRNA undergoes alternative exon splicing, a mechanism whereby multiple transcripts and proteins can be generated from a single gene, contributing to tissue-specific diversity in protein function.^21,22^ In the case of Myo5a, splicing could theoretically produce eight arrangements having constitutive exons A, C and E along with various assortments of the optional exons B, D, and F. With few exceptions, however, the functions of Myo5a splice variants are not well understood.

The effects of Myo5a deletion or mutation underscore the importance of this protein’s presence in sufficient mass amount and the appropriate regulation of its exon splicing. Indeed, the distinct roles of exons B and F in cargo transport were recognized through studies of Myo5a genetic defects. Reduced pigmentation is an obvious sign of *Myo5a* mutation and has been documented across species. However, the more devastating consequences of Myo5a deletion or mutation are neurological. Homozygosity for the *Myo5a* null allele is a fatal defect associated with opisthotonus and convulsions leading to premature death.^23–26^ In humans, *MYO5a* mutations cause the rare autosomal recessive disorder, Griscelli syndrome type I, in which hypopigmentation is coupled with cognitive impairment, delayed motor development, and hypotonia.^27^

Exons B and F are, respectively, hallmarks of the brain (neuron) and skin (melanocyte) variants, conferring the capacity to interact with key cargos (the enzyme n-Nos and the melanosome organelle, respectively).^11,13–17,28^ The interaction of exon F in the ABCDEF and ABCEF variants of Myo5a with Rab27a is likewise required in endothelial cells for transport of Weible-Palade bodies, the storage organelles for the hemostatic protein, von Willebrand factor.^29^ A quite distinct role, the facilitation of intracellular mRNA distribution, has also been ascribed to the ABCE variant.^30^ Such a role could be critical for the spatial and temporal control of protein synthesis in polarized cell types such as neurons/nerves, where the site of mRNA transcription in the nucleus is distant from the site of action of the encoded protein.

Less is currently known regarding the functional attributes of exon D, although variants including this exon are transcribed in many tissues.^31^ Exon D mediates Myo5a binding to Rab8a and Rab10,^31^ which are involved in complex intracellular trafficking patterns of membrane-bound entities. In adipocytes and skeletal muscle, these Rabs associate with Glut4-containing secretory vesicles, facilitating their motion to the plasma membrane in a Myo5a-dependent manner after insulin stimulation.^32–34^ Interestingly, an early study of melanocyte Myo5a expression intimated that a recombinant protein fragment corresponding to the variant having exon F alone interacted more avidly with Rab27a, than did the fragment having both exons D and F. These data suggest the intriguing possibility that inclusion of exon D modulates the function of exon F, or that these two variants serve slightly different, but still unknown, functional roles in a cellular context.^14^

In addition to this mechanism (where an alternative exon provides a binding platform for a particular accessory protein or cargo), the exon composition could potentially affect structural properties of Myo5a dimers, the functional form of this motor protein. The alternative exons of Myo5a are interspersed among coiled-coil segments that form a dimerization domain for Myo5a monomers. In brain Myo5a, the first and largest coiled-coil domain is located entirely N-terminal to exon A, while a second ends within it. The remainder of exon A, as well as exons B and C are located distal to these domains. The third and final putative coiled-coil domain of brain Myo5a encompasses all of exon E and extends into the GTD.^10^ It has been shown that binding of Dynll2 dimers to a recombinant fragment spanning this entire region structurally stabilizes Myo5a dimers.^11,12,15^ In contrast, insertion of exon F amino acid sequence between that of exon E and the GTD is anticipated to disrupt the third coiled-coil, dividing it into two segments and potentially exerting a negative influence on Myo5a three-dimensional structure or stability.^3,8,10^ Each optional exon is thus expected to influence the structure and support diverse functions, of Myo5a, although the subject is still incompletely understood.

In this report, we characterized Myo5a splice variants expressed in the murine stomach. In extracting each gastric region, all cell types were comingled. However, fluorescent in situ hybridization, a sensitive technique that maintains tissue structure while permitting visualization of a target gene’s mRNA expression in its native spatial context, indicated that pyloric Myo5a mRNA was concentrated within the myenteric plexus. There, Myo5a mRNA was in proximity to mRNA encoding βIII-Tubulin, a major component of microtubules which is highly expressed in neurons (Figure 2). The myenteric plexus, a network of enteric neurons and nerve-supporting cells which lies between the external longitudinal and internal circular smooth muscle layers along the entire gastrointestinal tract, was identified as the predominant site of Myo5a expression and thus was considered to be the source of the majority of the Myo5a mRNA analyzed in this study. Furthermore, Myo5a protein colocalized in the plexus with the neural marker, βIII-Tubulin, an intermediate filament protein expressed by nerves of the peripheral nervous system, and in filaments extending into the muscular layers with synaptophysin, a protein associated with synaptic vesicles in nerve termini (Figure 1).

Relative gene expression assays were performed, first to assess the extent of overall Myo5a mRNA expression in antrum, body, fundus, and pylorus, and then to compare expression of the alternate exons B and F. Subsequently, restriction analysis provided a means to determine the linear configurations of alternate exons in Myo5a transcripts and to further interrogate the transcripts for the presence of exon D. A complex mixture of splice variants was detected in all stomach regions.

We report that overall Myo5a mRNA expression was lower in pylorus than in antrum, fundus, or body, which expressed total Myo5a to approximately equivalent extents (Figure 3). Nitric oxide, produced at nerve termini by the enzyme n-Nos, is an inhibitory neurotransmitter in the gastrointestinal tract, along with ATP/other purines and perhaps peptides such as VIP (reviewed in Ref.^35^). The data presented here indicate that the extents of expression of Myo5a exon B and n-Nos were roughly proportional, and both were lower in pylorus than in the other stomach regions. Lower expression of n-Nos mRNA in pylorus compared with fundus/body and antrum has also been observed in rat stomach,^36^ suggesting that it is a physiological feature of this region. Regardless of the absolute extent of expression, however, the critical importance of nitric oxide to pyloric sphincter function has been amply demonstrated by the severe dysfunction observed consequent to restrictions of pyloric nitric oxide synthesis (for example, concomitant with n-Nos knock-out).^37^ Because the only form of Myo5a that can interact with the accessory protein Dynll2 and form a transport complex with n-Nos contains exon B,^6,12,38^ the data we present also demonstrate the critical importance of Myo5a, and specifically of exon B-containing Myo5a variant(s), to pyloric function. In pylorus and other stomach regions, our data further indicate that exon B was found almost exclusively within the exon configuration ABCE (Figures 4 and 5).

Restriction analyses indicated that transcripts including exon D were more prevalent in fundus and pylorus than in antrum or body, although they were represented to some extent in each region. Expression of exon F was also detected throughout the stomach, with its relative abundance greatest in the fundus and antrum. Considering the lower overall Myo5a expression of pylorus, it was striking that exon F expression in this region nearly equaled that of the other regions, suggesting that exon F-containing variants were particularly abundant in pylorus. Notably, no evidence was found that exon B occurred in the same transcripts with either exon D or exon F. Conversely, most exon F expressed was within transcripts that also included exon D. Taken together, these data suggest that gastric tissues express several different splice variants of Myo5a, with the arrangements ABCE, ACDEF, and ACEF positively identified.

This initial molecular study has demonstrated the presence of an array of Myo5a splice variants at the mRNA level, which differed in relative expression and/or constitution across the murine stomach. Since the splice variants of this motor protein that are expressed, and the ratios among them, are likely to be adapted to transport demands particular to each region, subtle alterations of Myo5a variant expression may be relevant to neurotransmission and may carry broader implications for Myo5a-dependent neurotransmission in visceral organs.

## Supplementary Material

Supplemental Figures

Additional supporting information may be found online in the Supporting Information section.

## Figures and Tables

**FIGURE 1:**
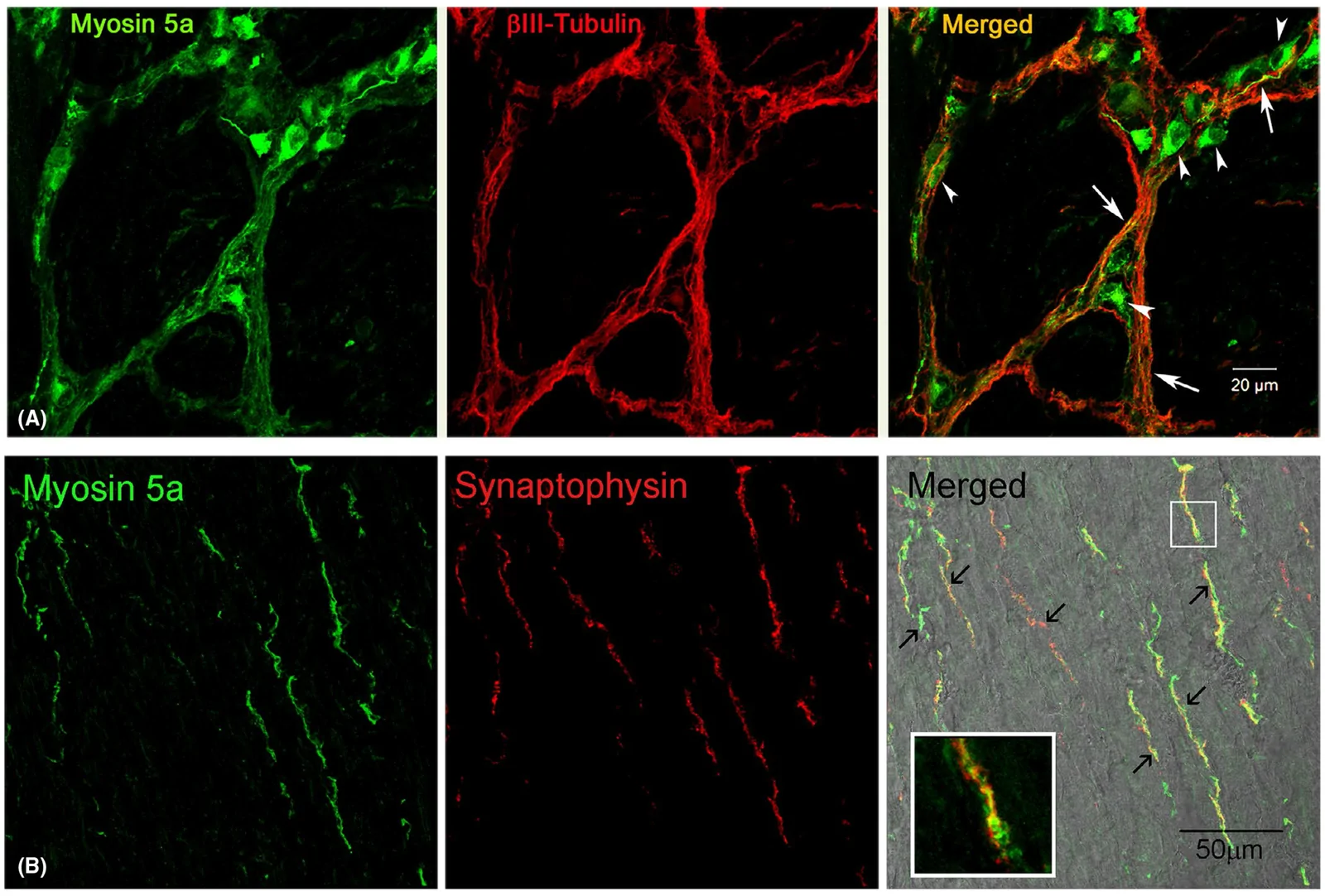
Photomicrographs of Myosin 5a in the stomach. (Panel A) LMMP preparation from the murine gastric fundus double labeled with Myo5a (green channel) and neuronal-specific βIII-Tubulin (red channel). Abundant Myo5a immunoreactivity was detected in neuronal processes (arrows) and perikarya (arrowheads) within the myenteric plexus. Extensive colocalization between Myo5a and βIII-Tubulin immunoreactivity is shown in the merged panel. Projection images in each panel are made from seven optical slices acquired at 0.55 μm intervals (40×). Scale bar = 20 μm. (Panel B) Double label confocal microscopy images (40×) of transverse sections of mouse pylorus showing positive immunoreactivity for Myo5a (green channel) and synaptophysin (red channel). The colocalization between these proteins (yellow) is shown in the merged panel on the right, which is overlaid on the differential interference contrast image to highlight tissue structure. Nerve fibers are indicated by the arrows. The inset (63×) shows co-localization of Myo5a and synaptophysin within the circular smooth muscle layer (scale bar = 50 μm)

**FIGURE 2:**
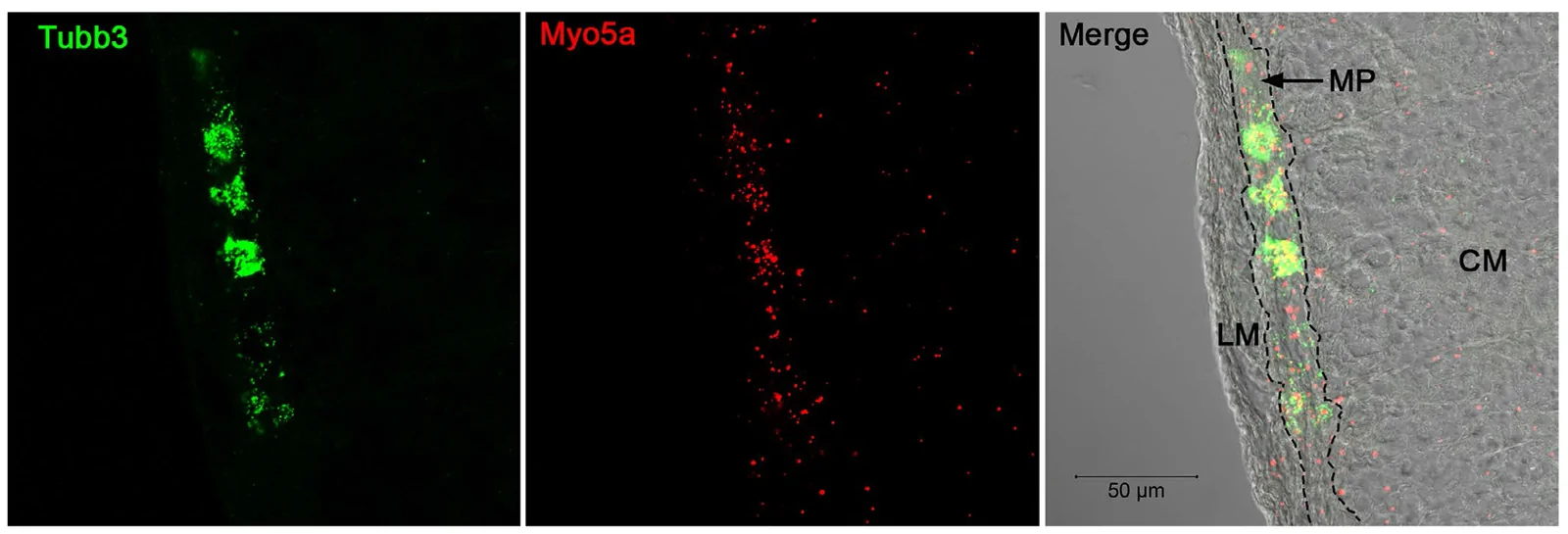
In situ hybridization of murine pylorus. Fluorescent in situ hybridization of pylorus, showing longitudinal and circular muscle layers (LM, CM) separated by myenteric plexus (MYP; between dashed lines). The expression of βIII-Tubulin mRNA (Tubb3) is indicated by the green punctae, and of all variants of Myo5a mRNA by the red punctae. Magnification 40×; scale bar = 50 μm

**FIGURE 3:**
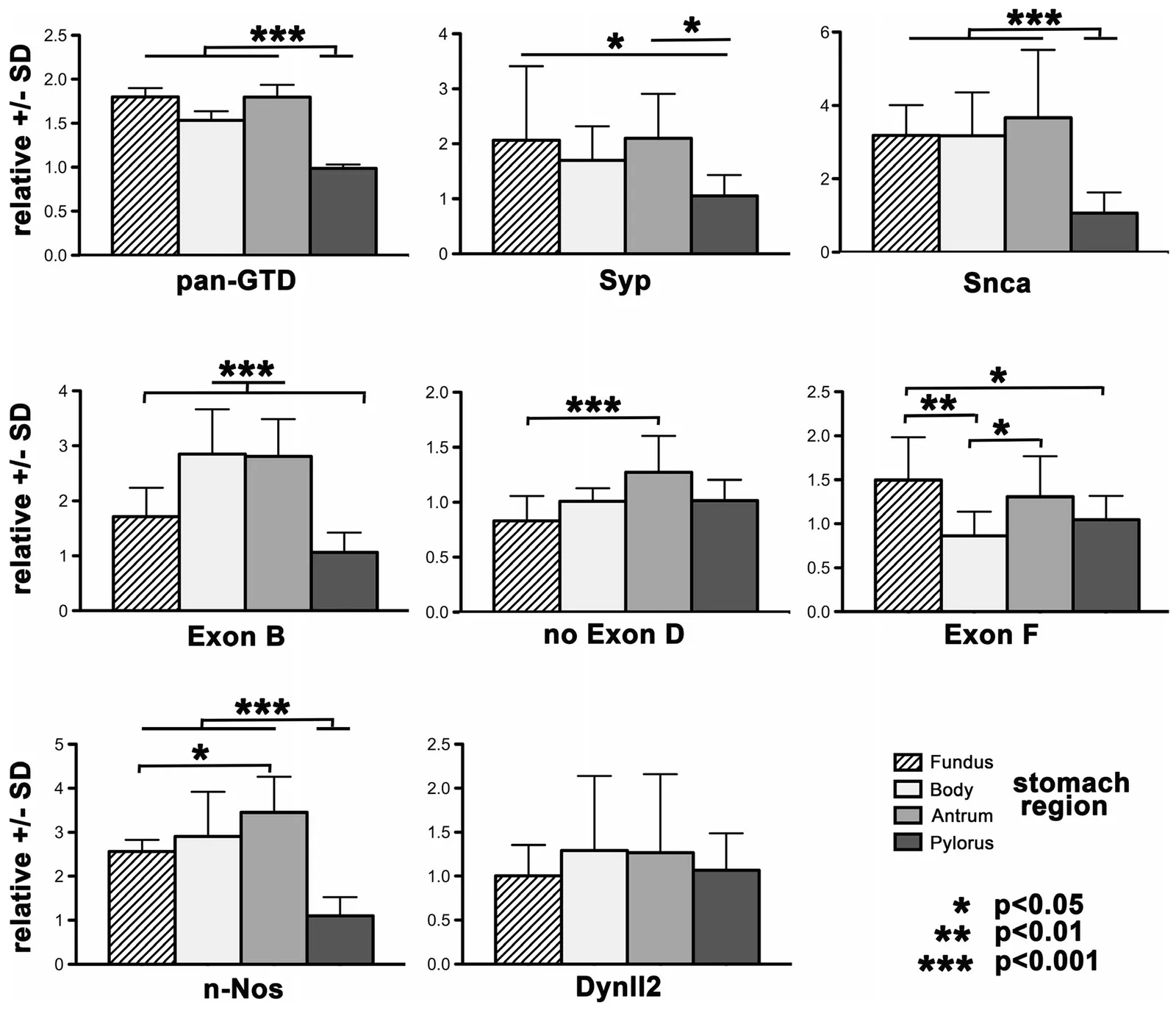
Real-time PCR of stomach regions. cDNA prepared from total RNA of stomach sections was assessed for Myo5a mRNA expression, using a pan-assay detecting all splice variants, as well as by assays reporting on the presence/absence of exons B, D and F. The expression of known Myo5a interactive partners (n-Nos and Dynll2), and of nerve terminus markers (Syp and Snca) were also measured, as was the internal control, 18S rRNA. Antrum and pylorus samples from three animals, and fundus and pylorus samples from four animals, were tested in triplicate for each amplicon. The average relative extents of expression for antrum, body, and fundus were graphed ±SD, in comparison to pylorus. The amplicon tested is shown below each graph. Comparisons that were significant by ANOVA are indicated (*p < 0.5; **p < 0.1; ***p < 0.001)

**FIGURE 4:**
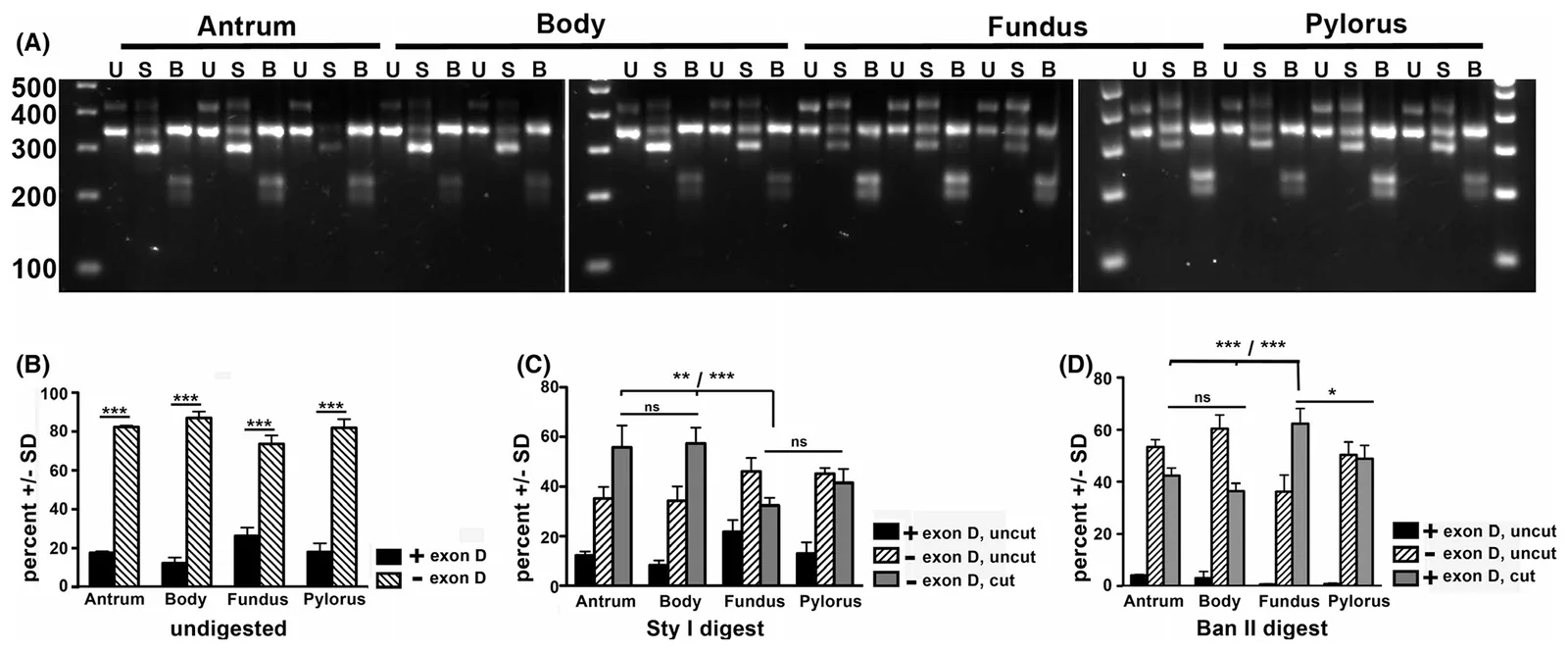
Stomach Myo5a variants include alternative exons B and D. cDNA was used as the template for initial PCR with primer pair 1, followed by nested PCR with primer pair 2. The products of the nested reaction were analyzed by agarose gel electrophoresis either undigested (U), or after digestion with Sty I (S) or Ban II (B). Panel A shows electrophoresis of fourteen samples (three antrum, four body, four fundus and three pylorus) under each condition, in comparison to a 100 bp ladder in the first lane of each gel and the final lane at the far right, for estimation of mass. Panel B shows graphic representation of electrophoresis results, with bars indicating the average percentage of material quantitated in each band per lane ±SD. The abundance of each band observed in the undigested lanes (panel B), the Sty I digestion lanes (panel C) and the Ban II digestion lanes (panel D) are grouped according to stomach region. Comparisons that were significant by ANOVA are shown (*p < 0.5; **p < 0.1; ***p < 0.001), as are key non-significant comparisons (ns)

**FIGURE 5:**
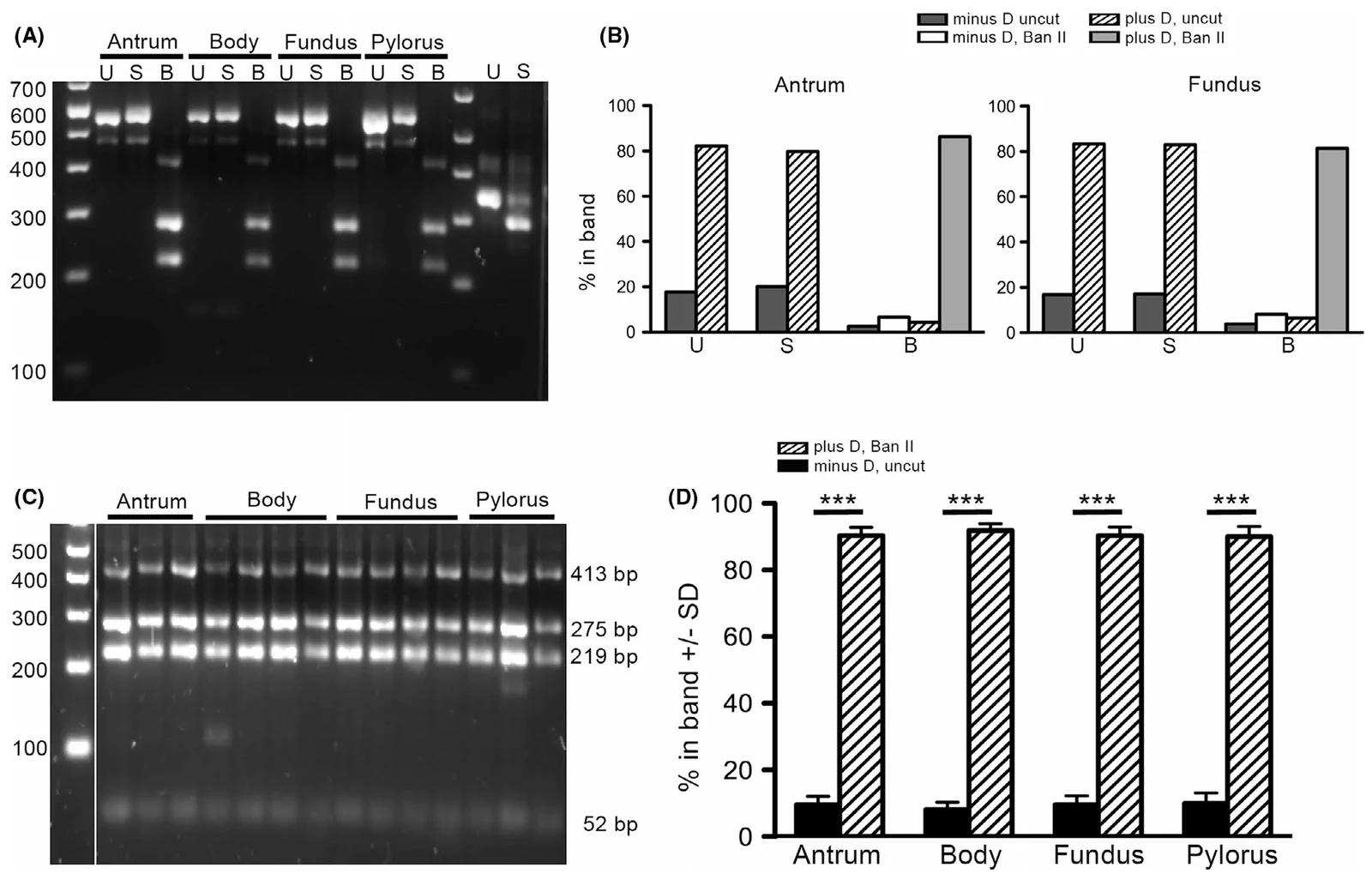
Contiguous expression of exons D and F in gastric Myo5a splice variants. cDNA was used as the template for initial PCR with primer pair 1, followed by nested PCR with primer pair 3, and products were electrophoresed either undigested (U), or following digestion with Sty I (S) or Ban II (B). A 100 bp DNA ladder was run in an adjacent lane for mass estimation. Panel A shows the electrophoresis pattern obtained from a single animal: Ban II cleaved the larger product while Sty I cleaved neither product, a pattern that was consistent across stomach regions. The lanes at the far right show Sty I digestion performed concurrently on an antrum PCR done with primer pair 2, which was included to demonstrate Sty I enzymatic activity under the conditions employed. Panel B shows the graphic representation of the data for the virtually identical antrum and fundus samples from panel A, which indicated that neither PCR product was susceptible to Sty I cleavage and therefore did not include exon B. Both products were completely cleaved by Ban II, because a common Ban II site is present in all fragments made by primer pair 3 PCR. The minor digestion product of 413 bp is predicted to arise from the lower abundance PCR product lacking exon D. However, the 275 bp/219 bp fragments can be generated by Ban II cleavage only of PCR products encompassing exon D. In panel C, the Ban II digestion patterns for multiple regions (three antrum, four body, four fundus and three pylorus) are shown, with 100 bp ladder in the first lane. The white line indicates where an intervening lane has been removed from the image. The percentage per lane ±SD of the minus exon D versus plus exon D products from this gel are graphically presented in panel D. Comparisons between groups that were significant by ANOVA are shown (***p < 0.001)

## Keypoints

- Myosin 5a (Myo5a) is known to be expressed highly in cells specialized for secretion, including brain neurons and melanocytes, which express distinct splice variants of this motor protein suited for interactions with tissue-specific accessory proteins and secretory cargo. However, the Myo5a splice variants expressed by peripheral nerves have not been extensively studied.
- This study examined Myo5a expression across the murine stomach. Myo5a mRNA and protein were identified in the myenteric plexus, and Myo5a protein was found at nerve termini within the muscular layers. Molecular analyses indicated that multiple splice variants of Myo5a mRNA were expressed and differentially distributed in antrum/body versus fundus/pylorus regions.
- The splice variants expressed are expected to reflect cargo transport requirements important for Myo5a-dependent neurotransmission roles in different stomach regions.

## References

[1] Wollert T, Patel A, Lee YL, Myosin5a tail associates directly with Rab3A-containing compartments in neurons. J Biol Chem. 2011;286(16):14352–14361. 10.1074/jbc.M110.187286 PMC3077635 21349835

[2] Prekeris R, Terrian DM. Brain myosin V is a synaptic vesicle-associated motor protein: evidence for a Ca2+-dependent interaction with the synaptobrevin-synaptophysin complex. J Cell Biol. 1997;137(7):1589–1601. 10.1083/jcb.137.7.1589 PMC2137828 9199173

[3] Watanabe M, Nomura K, Ohyama A, Myosin-Va regulates exocytosis through the submicromolar Ca2+-dependent binding of syntaxin-1A. Mol Biol Cell. 2005;16(10):4519–4530. 10.1091/mbc.E05-03-0252 PMC1237061 16030255

[4] Buttow NC, Espreafico EM, de Souza RR, Romano EB. Immunolocalization of myosin-V in the peribronchial, intrapulmonary peritracheal plexuses of the Wistar rat. J Neurosci Methods. 2006;152(1-2):274–277. 10.1016/j.jneumeth.2005.09.012 16271276

[5] Drengk AC, Kajiwara JK, Garcia SB, Immunolocalisation of myosin-V in the enteric nervous system of the rat. J Auton Nerv Syst. 2000;78(2-3):109–112. 10.1016/s0165-1838(99)00073-9 10789689

[6] Chaudhury A, He XD, Goyal RK. Myosin Va plays a key role in nitrergic neurotransmission by transporting nNOSalpha to enteric varicosity membrane. Am J Physiol Gastrointest Liver Physiol. 2011;301(3):G498–G507. 10.1152/ajpgi.00164.2011 PMC3174543 21680773

[7] Chaudhury A, Cristofaro V, Carew JA, Goyal RK, Sullivan MP. Myosin Va plays a role in nitrergic smooth muscle relaxation in gastric fundus and corpora cavernosa of penis. PLoS One. 2014;9(2):e86778. 10.1371/journal.pone.0086778 PMC3916320 24516539

[8] Seperack PK, Mercer JA, Strobel MC, Copeland NG, Jenkins NA. Retroviral sequences located within an intron of the dilute gene alter dilute expression in a tissue-specific manner. EMBO J. 1995;14(10):2326–2332. 10.1002/j.1460-2075.1995.tb07227.x PMC398341 7774591

[9] Lambert J, Naeyaert JM, Callens T, De Paepe A, Messiaen L. Human myosin V gene produces different transcripts in a cell type-specific manner. Biochem Biophys Res Commun. 1998;252(2):329–333. 10.1006/bbrc.1998.9644 9826529

[10] Au JS, Huang JD. A tissue-specific exon of myosin Va is responsible for selective cargo binding in melanocytes. Cell Motil Cytoskeleton. 2002;53(2):89–102. 10.1002/cm.10061 12211107

[11] Wagner W, Fodor E, Ginsburg A, Hammer JA 3rd. The binding of DYNLL2 to myosin Va requires alternatively spliced exon B and stabilizes a portion of the myosin’s coiled-coil domain. Biochemistry. 2006;45(38):11564–11577. 10.1021/bi061142u 16981716

[12] Hodi Z, Nemeth AL, Radnai L, Alternatively spliced exon B of myosin Va is essential for binding the tail-associated light chain shared by dynein. Biochemistry. 2006;45(41):12582–12595. 10.1021/bi060991e 17029413

[13] Wu X, Wang F, Rao K, Sellers JR, Hammer JA 3rd. Rab27a is an essential component of melanosome receptor for myosin Va. Mol Biol Cell. 2002;13(5):1735–1749. 10.1091/mbc.01-12-0595 PMC111140 12006666

[14] Westbroek W, Lambert J, Bahadoran P, Interactions of human Myosin Va isoforms, endogenously expressed in human melanocytes, are tightly regulated by the tail domain. J Invest Dermatol. 2003;120(3):465–475. 10.1046/j.1523-1747.2003.12068.x 12603861

[15] Bodor A, Radnai L, Hetenyi C, DYNLL2 dynein light chain binds to an extended linear motif of myosin 5a tail that has structural plasticity. Biochemistry. 2014;53(45):7107–7122. 10.1021/bi500574z 25312846

[16] Li XD, Ikebe R, Ikebe M. Activation of myosin Va function by melanophilin, a specific docking partner of myosin Va. J Biol Chem. 2005;280(18):17815–17822. 10.1074/jbc.M413295200 15760894

[17] Fukuda M, Kuroda TS, Mikoshiba K. Slac2-a/melanophilin, the missing link between Rab27 and myosin Va: implications of a tripartite protein complex for melanosome transport. J Biol Chem. 2002;277(14):12432–12436. 10.1074/jbc.C200005200 11856727

[18] Espindola FS, Suter DM, Partata LB, The light chain composition of chicken brain myosin-Va: calmodulin, myosin-II essential light chains, and 8-kDa dynein light chain/PIN. Cell Motil Cytoskeleton. 2000;47(4):269–281. 10.1002/1097-0169(200012)47:4<269::AID-CM2>3.0.CO;2-G 11093248

[19] Jaffrey SR, Snyder SH. PIN: an associated protein inhibitor of neuronal nitric oxide synthase. Science. 1996;274(5288):774–777. 10.1126/science.274.5288.774 8864115

[20] Rapali P, Szenes A, Radnai L, Bakos A, Pal G, Nyitray L. DYNLL/LC8: a light chain subunit of the dynein motor complex and beyond. FEBS J. 2011;278(17):2980–2996. 10.1111/j.1742-4658.2011.08254.x 21777386

[21] Johnson JM, Castle J, Garrett-Engele P, Genome-wide survey of human alternative pre-mRNA splicing with exon junction microarrays. Science. 2003;302(5653):2141–2144. 10.1126/science.1090100 14684825

[22] Zavolan M, Kondo S, Schonbach C, Impact of alternative initiation, splicing, and termination on the diversity of the mRNA transcripts encoded by the mouse transcriptome. Genome Res. 2003;13(6B):1290–1300. 10.1101/gr.1017303 PMC403716 12819126

[23] Brooks SA, Gabreski N, Miller D, Whole-genome SNP association in the horse: identification of a deletion in myosin Va responsible for Lavender Foal Syndrome. PLoS Genet. 2010;6(4):e1000909. 10.1371/journal.pgen.1000909 PMC2855325 20419149

[24] Futaki S, Takagishi Y, Hayashi Y, Identification of a novel myosin-Va mutation in an ataxic mutant rat, dilute-opisthotonus. Mamm Genome. 2000;11(8):649–655. 10.1007/s003350010121 10920234

[25] Landrock KK, Sullivan P, Martini-Stoica H, Pleiotropic neuropathological and biochemical alterations associated with Myo5a mutation in a rat Model. Brain Res. 2018;1679:155–170. 10.1016/j.brainres.2017.11.029 PMC7696654 29217155

[26] Mercer JA, Seperack PK, Strobel MC, Copeland NG, Jenkins NA. Novel myosin heavy chain encoded by murine dilute coat colour locus. Nature. 1991;349(6311):709–713. 10.1038/349709a0 1996138

[27] Griscelli C, Durandy A, Guy-Grand D, Daguillard F, Herzog C, Prunieras M. A syndrome associating partial albinism and immunodeficiency. Am J Med. 1978;65(4):691–702. 10.1016/0002-9343(78)90858-6 707528

[28] Espindola FS, Espreafico EM, Coelho MV, Biochemical and immunological characterization of p190-calmodulin complex from vertebrate brain: a novel calmodulin-binding myosin. J Cell Biol. 1992;118(2):359–368. 10.1083/jcb.118.2.359 PMC2290054 1378447

[29] Rojo Pulido I, Nightingale TD, Darchen F, Seabra MC, Cutler DF, Gerke V. Myosin Va acts in concert with Rab27a and MyRIP to regulate acute von-Willebrand factor release from endothelial cells. Traffic. 2011;12(10):1371–1382. 10.1111/j.1600-0854.2011.01248.x 21740491

[30] Salerno VP, Calliari A, Provance DW Jr, Sotelo-Silveira JR, Sotelo JR, Mercer JA. Myosin-Va mediates RNA distribution in primary fibroblasts from multiple organs. Cell Motil Cytoskeleton. 2008;65(5):422–433. 10.1002/cm.20272 PMC2633925 18357619

[31] Roland JT, Lapierre LA, Goldenring JR. Alternative splicing in class V myosins determines association with Rab10. J Biol Chem. 2009;284(2):1213–1223. 10.1074/jbc.M805957200 PMC2613619 19008234

[32] Yoshizaki T, Imamura T, Babendure JL, Lu JC, Sonoda N, Olefsky JM. Myosin 5a is an insulin-stimulated Akt2 (protein kinase Bbeta) substrate modulating GLUT4 vesicle translocation. Mol Cell Biol. 2007;27(14):5172–5183. 10.1128/MCB.02298-06 PMC1951956 17515613

[33] Sun Y, Chiu TT, Foley KP, Bilan PJ, Klip A. Myosin Va mediates Rab8A-regulated GLUT4 vesicle exocytosis in insulin-stimulated muscle cells. Mol Biol Cell. 2014;25(7):1159–1170. 10.1091/mbc.E13-08-0493 PMC3967978 24478457

[34] Chen Y, Wang Y, Zhang J, Rab10 and myosin-Va mediate insulin-stimulated GLUT4 storage vesicle translocation in adipocytes. J Cell Biol. 2012;198(4):545–560. 10.1083/jcb.201111091 PMC3514028 22908308

[35] Curro D, Ipavec V, Preziosi P. Neurotransmitters of the non-adrenergic non-cholinergic relaxation of proximal stomach. Eur Rev Med Pharmacol Sci. 2008;12(Suppl 1):53–62. 18924444

[36] Saur D, Neuhuber WL, Gengenbach B, Huber A, Schusdziarra V, Allescher HD. Site-specific gene expression of nNOS variants in distinct functional regions of rat gastrointestinal tract. Am J Physiol Gastrointest Liver Physiol. 2002;282(2):G349–G358. 10.1152/ajpgi.00226.2001 11804857

[37] Huang PL, Dawson TM, Bredt DS, Snyder SH, Fishman MC. Targeted disruption of the neuronal nitric oxide synthase gene. Cell. 1993;75(7):1273–1286. 10.1016/0092-8674(93)90615-w 7505721

[38] Chaudhury A, Rao YM, Goyal RK. PIN/LC8 is associated with cytosolic but not membrane-bound nNOS in the nitrergic varicosities of mice gut: implications for nitrergic neurotransmission. Am J Physiol Gastrointest Liver Physiol. 2008;295(3):G442–G451. 10.1152/ajpgi.90280.2008 PMC2536782 18635601

