# Supplementary material for: Expression of Myosin 5a splice variants in murine stomach
Source: Neurogastroenterol Motil. Author manuscript; Available in PMC 2026 Nov 16. (PMC13772183; doi:10.1111/nmo.14162)
Supplement: Supplementary file 1 [file NIHMS2219333-supplement-Supplemental_Figures.pdf]

SUPPORTING INFORMATION

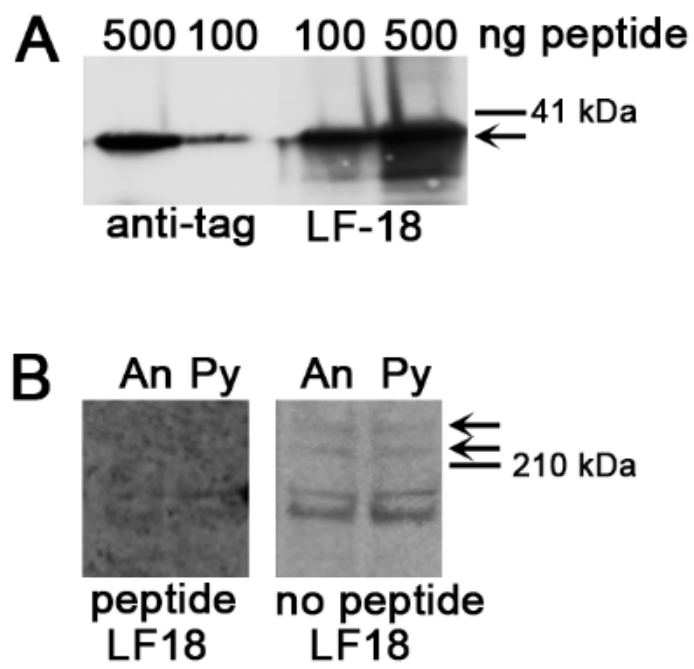

Supplemental Figure 1. Specificity of anti-Myo5a Antibody. Aliquots of recombinant peptide (residues 1531-1855 of human Myo5a with N-terminal hexahistidine tag expressed in E. coli (LSG15292/183344, LS-Bio, Seattle WA, USA) were electrophoresed on a 3-8% Tris-acetate gel, transferred to a nitrocellulose membrane using the iBlot semi-dry system (Thermo-Fisher Scientific, Waltham MA USA), blocked in 5% dry milk in Tris buffered saline plus 1% Tween-20, and probed overnight with antibody either to tag (LS Bio; C157807, 1:1000) or to Myo5a (Sigma-Aldrich , St Louis MO USA; M4812 LF-18, 1:1000), then incubated with HRP-conjugated secondary antibodies (1:10,000) for 2 hr at room temperature. Blots were developed with Enlightening ECL reagents (PerkinElmer, Waltham MA, USA) and imaged on an Amersham Imager 600 (General Electric, Pittsburgh PA, USA) at CCD resolution 2048x1536, using Imager 600 software. Extracts of C57/Bl6 antrum and pylorus were prepared in RIPA buffer supplemented with protease and phosphatase inhibitors (Santa Cruz Biotechnology, Santa Cruz, CA USA) in the Tissue Lyser II (Qiagen, Gaithersburg, MD USA) for 2 x 3 min at 30 Hz, then centrifuged at 14000 rpm for 10 min at 4°C to pellet insoluble material. Protein concentration of the supernatant was determined by BCA assay (Sigma-Aldrich). 5 μg extract was electrophoresed per lane, transferred and blocked, then incubated with either LF-18 antibody alone, or antibody preincubated with Myo5a peptide, and handled as described above.

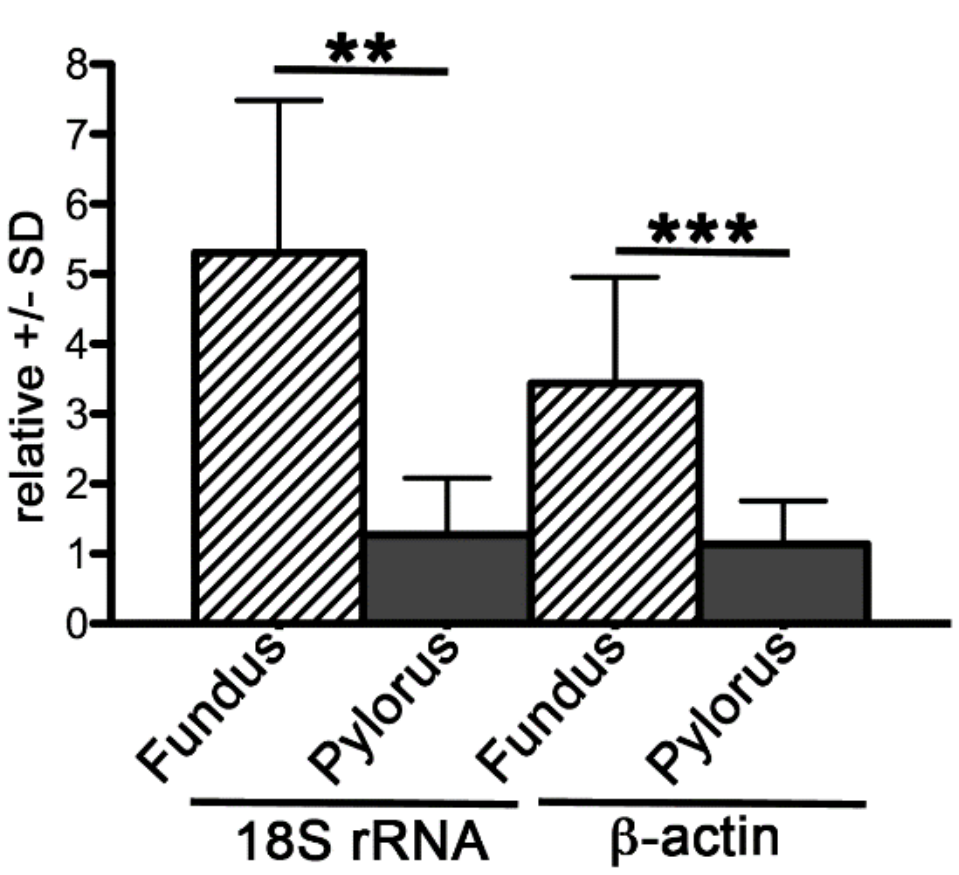

Supplemental Figure 2. Comparison of Endogenous Controls for RT-PCR. Fundus and pylorus tissues from three C57/Bl6 animals were stabilized by immersion in RNA-later solution (Sigma-Aldrich). Total RNA was purified using the Qiagen RNeasy Plus Mini kit, following tissue

disruption in buffer RLT plus ß-mercaptoethanol with a 5mm stainless steel bead, by two cycles of three minutes each at 30Hz in the Tissue Lyser II apparatus. Total RNA concentration and purity were determined by ultraviolet spectrophotometry. Aliquots of total RNA were converted to cDNA using the SuperScript IV VILO mastermix, then used for triplicate reverse-transcriptase realtime PCR (RT-PCR) with universal PCR mastermix and TaqMan primer/probe sets for an exon/exon boundary within the GTD of murine Myo5a (mM01146045_m1) and for alternate internal controls (18S ribosomal RNA, Hs99999901_s1; or beta actin, Mm01205647_m1). All reagents for RTPCR were from Thermo-Fisher Scientific. Reactions were done on an Applied Biosystems QuantStudio 6 Flex instrument under a standard protocol (40 amplification cycles of 15 sec at 94ºC and 60 sec at 60ºC) in triplicate, normalized to triplicate internal control determinations, and analyzed by the ΔΔCt method. Control assays were performed without added cDNA.
